# Intratumoral immunotherapy of murine pheochromocytoma shows no age-dependent differences in its efficacy

**DOI:** 10.3389/fendo.2023.1030412

**Published:** 2023-05-08

**Authors:** Ondrej Uher, Katerina Hadrava Vanova, Radka Lencova, Andrea Frejlachova, Herui Wang, Zhengping Zhuang, Jan Zenka, Karel Pacak

**Affiliations:** ^1^ Section on Medical Neuroendocrinology, Eunice Kennedy Shriver National Institute of Child Health and Human Development, National Institutes of Health (NIH), Bethesda, MD, United States; ^2^ Department of Medical Biology, Faculty of Science, University of South Bohemia, Ceske Budejovice, Czechia; ^3^ Neuro-Oncology Branch, National Cancer Institute, National Institutes of Health (NIH), Bethesda, MD, United States

**Keywords:** intratumoral immunotherapy, age, pheochromocytoma, TLR ligands, pancreatic adenocarcinoma, anti-CD40 antibody

## Abstract

Cancer immunotherapy has shown remarkable clinical progress in recent years. Although age is one of the biggest leading risk factors for cancer development and older adults represent a majority of cancer patients, only a few new cancer immunotherapeutic interventions have been preclinically tested in aged animals. Thus, the lack of preclinical studies focused on age-dependent effect during cancer immunotherapy could lead to different therapeutic outcomes in young and aged animals and future modifications of human clinical trials. Here, we compare the efficacy of previously developed and tested intratumoral immunotherapy, based on the combination of polysaccharide mannan, toll-like receptor ligands, and anti-CD40 antibody (MBTA immunotherapy), in young (6 weeks) and aged (71 weeks) mice bearing experimental pheochromocytoma (PHEO). The presented results point out that despite faster growth of PHEO in aged mice MBTA intratumoral immunotherapy is effective approach without age dependence and could be one of the possible therapeutic interventions to enhance immune response to pheochromocytoma and perhaps other tumor types in aged and young hosts.

## 1 Introduction

Undoubtedly, the incidence of various cancers is increasing with age (1). The process of immune dysfunction that occurs with age, so-called immunosenescence, is characterized by increased autoimmune diseases, decreased adaptive immune response, and increased risk of infections (2). It has also been hypothesized that age-related immune system dysfunction can decrease cancer immunosurveillance and lead to immunosurveillance escape and tumorigenesis. Despite of increasing interest in intratumoral immunotherapies and age-associated differences in immune system function, there is limited number of studies that have been focused on these differences in aged mice and humans. The lack of age-related research and clinical trials that include aged patients could lead to the failure of new treatment approaches in those patients (3–5).

MBTA immunotherapy is based on intratumoral injection of mannan-BAM, polysaccharide from *Saccharomyces cerevisiae via* with a biocompatible anchor for cell membrane (BAM), a mixture of toll-like receptor (TLR) ligands, and anti-CD40 antibody. After the intratumoral injection, mannan-BAM anchors to the tumor cell surface via the BAM anchor and serves as a ligand stimulating phagocytosis via the activation of the complement lectin pathway and tumor cell opsonization (6–8). Mixture of TLR ligands, namely resiquimod, polyinosinic-polycytidylic acid, and lipoteichoic acid, supports the infiltration of immune cells into tumors, as well as their activation (9–11). Anti-CD40 is an agonistic monoclonal antibody that mimics the CD40 ligand expressed on helper T cells. After the ligation of CD40 expressed on antigen-presenting cell, such as macrophages, dendritic cells, and B cells, anti-CD40 leads to the activation of these cells, enhances antigen presentation, and induces an effective T cell anti-tumor response (12, 13). MBTA therapy has been previously tested in various subcutaneous tumor models in mice, such as melanoma (B16-F10), pancreatic adenocarcinoma (Panc02), colon carcinoma (CT26), and pheochromocytoma (PHEO, MTT cells) with the complete elimination of tumors in 62-83% of mice depending on tumor type (14–16). MBTA therapy has also shown systemic immune response in PHEO models including its metastatic model, bilateral Panc02, and bilateral CT26 model, where the therapy resulted in slower progression of non-treated distal tumors and significant prolongation of survival of treated mice (14, 16, 17). Moreover, strong infiltration of both adaptive and innate immune cells was observed in treated and distal tumors in case of bilateral PHEO model (18).

PHEO are rare catecholamine-producing neuroendocrine tumors derived from neural crest cells (19). These tumors are most common between the age 30-50 depending on catecholamine phenotypes and genetic background (20), but about 10-20% of all cases are diagnosed in pediatric patients (21). All the aforementioned studies focused on intratumoral MBTA therapy in murine PHEO have been performed in 6-8 weeks old mice which is equivalent to human 11-16 years of age (22). Therefore, in the present study, we assessed the efficacy of MBTA therapy in 6-weeks and 71-weeks old mice, which correlates to 11 and 56 years of human age, to evaluate possible age-dependent difference and more simulate a situation in patients with PHEO. To confirm our results on different tumor model, we also tested MBTA therapy in aged and young mice bearing bilateral Panc02 tumor model which more mimics the real situation with higher tumor burden.

## 2 Materials and methods

### 2.1 PHEO cell line, mice, tumor model establishment, and tumor size evaluation

Mouse tumor tissue-derived (MTT) cells, used in this study, are rapidly growing cells derived from liver metastases of mouse pheochromocytoma (MPC) cells (23, 24). Cells were maintained in Dulbecco’s modified eagle media (DMEM) (Sigma-Aldrich, Saint Lous, MO, USA) supplemented with 10% heat-inactivated fetal bovine serum (Gemini, West Sacramento, CA, USA), and 100 U/mL of penicillin/streptomycin (Gibco; Thermo Fisher Scientific, Waltham, MA, USA). Cells were cultured at 37 °C in humidified air with 5% CO_2_. Cell lines was tested for mycoplasma using the MycoAlert™ detection kit purchased from Lonza (Walkersville, MD, USA). Both female young (6 weeks) and aged (71 weeks) C57BL/6J mice were purchased from Jackson Laboratory (Bar Harbor, ME, USA). For establishment of PHEO tumors, mice were subcutaneously injected in the previously shaved right lower dorsal site with 3 *×* 10^6^ MTT cells in 0.2 mL of DMEM without additives. For rechallenge experiment, the mice were s.c. injected in the previously shaved left lower dorsal site (opposite site) with the same number of MTT cells as mentioned above.

Tumor volume was measured every fourth day with a caliper and calculated as V = (π/6) AB^2^ (A and B, the largest and the smallest dimension of tumor, respectively). Survival curves are based on (1): the time of death of experimental mice, (2) the time of euthanasia of experimental mice reaching the maximal allowed tumor volume 2000 mm^3^, (3) tumor diameter exceeding 2 cm, (4) non-healing skin necrosis over the tumor. Materials and methods for Panc02 model are described in **Supplementary Material**.

### 2.2 MBTA therapy

Mannan from *Saccharomyces cerevisiae*, lipoteichoic acid (LTA) from *Bacillus subtilis*, and polyinosinic:polycytidylic acid (poly(I:C)) was obtained from Sigma-Aldrich (Saint Lous, MO, USA). BAM was obtained from NOF Corporation (White Plains, NY, USA). Resiquimod (R-848) was obtained from Tocris Bioscience (Minneapolis, MN, USA). Monoclonal anti-CD40 (clone FGK4.5/FGK45) was obtained from BioXCell (West Lebanon, NH, USA). Mannan-BAM synthesis was performed as previous reported (25). After development of tumors (average tumor volume 55 mm^3^), mice were treated intratumorally in days 0, 1, 2, 8, 9, 10, 16, 17, 18, 24, 25, and 26 with 50 μL of the therapeutic mixture consisting of 0.5 mg R-848 (HCl form), 0.5 mg poly(I:C), 0.5 mg LTA, and 0.4 mg anti-CD40 per mL of 0.2 mM mannan-BAM in PBS (MBTA therapy).

### 2.3 Real-time polymerase chain reaction

Pheochromocytoma samples (n=6/group) were dissected on day 46 after subcutaneous transplantation of tumor cells. Total RNA was extracted using PureLink RNA miniKit (Invitrogen, Walham, MA) and 1 ug of RNA was converted to cDNA using High-capacity cDNA Reverse Transcription Kit (Applied Biosystem, Walham, MA) according to manufacturer’s instructions. Real-time PCR was performed on the ViiA™ 7 System (Applied Biosystems, Carlsbad, CA) using the PowerUp SYBR Green Master Mix (Applied Biosystem) with recommended standard cycling mode by manufacturer. Each sample was run in doublet. The 2^–ΔΔCt^ was used to calculate relative gene expression for *Cd3e*, *Cd4*, *Cd8a*, and *Cd68* after normalization to the *Gapdh* internal control. Primer sequences of targeted genes are listed in **Supplementary Table 1**.

### 2.4 Immunohistochemistry

Paraffin-embedded tumor specimens were cut into 5-μm sections, and tissue slides were deparaffinized with Histo-Clear (National Diagnostics, Atlanta, GA, USA) and rehydrated by sequential incubation in ethanol at different concentrations. Antigen retrieval was performed by incubation of the slides in boiling citrate buffer for 20 min, followed by incubation with 3% H_2_O_2_ and blocking buffer (Sigma-Aldrich, St. Louis, MO, USA). The slides were probed with rabbit anti-CD3 antibody [SP7] (ab16669; dilution 1:150; Abcam, Cambridge, UK) or rabbit anti-CD45 antibody [D3F8Q] (70257S; dilution 1:200, Cell Signaling Technology, Danvers, MA, USA) overnight at 4°C. The signal was amplified using Anti-Rabbit EnVision+ System, HRP (Dako, Carpinteria, CA, USA) for 1h at room temperature and visualized using the liquid DAB+ Substrate Chromogen System (Dako). Sections were viewed at 10× magnification using a microscope BZ-X710 (Keyence Corporation, Osaka, Japan). The positively stained cells were counted in three randomly selected field of view by BZ-X Analyzer (1.3.1.1., Keyence Corporation) software and the mean for each sample was calculated.

### 2.5 Data analysis and statistics

For analyses of tumor growth and reduction during MBTA therapy, the area under the curve (AUC) was calculated, and statistical analysis was performed on AUC values using Two-way ANOVA. Kaplan-Meier survival curves were compared using a Log-rank test. Data were analyzed using GraphPad Prism version 8 (GraphPad Software, La Jolla, CA, USA) and STATISTICA 12 (StatSoft, Inc., Tulsa, OK, USA). Error bars indicate the standard error of the mean (SEM).

## 3 Results

### 3.1 Growth of subcutaneous murine pheochromocytoma (PHEO) in young and aged mice

**Figure 1:**
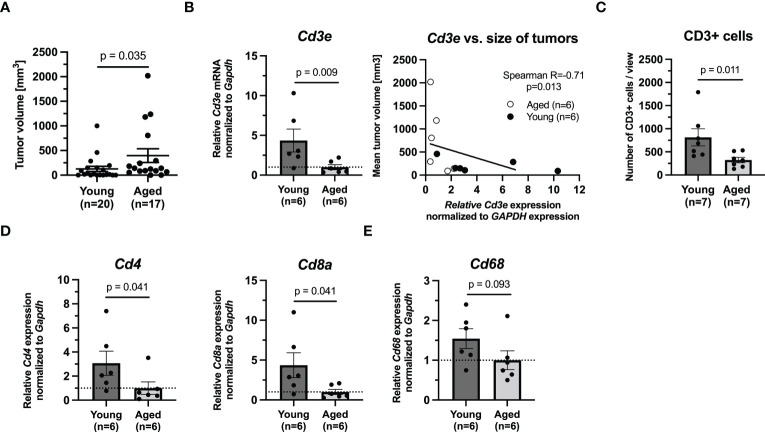
The growth of tumors in (6 weeks) and aged (71 weeks) mice bearing PHEO tumor. **(A)** The tumor volume in young and aged mice after 46 days. **(B)** mRNA levels of *Cd3e* and correlation analysis between *Cd3e* and size of tumor. **(C)** Immunohistochemical analysis of CD3 in tumors. **(D)** mRNA levels of *Cd4* and *Cd8a* as representative of T cells populations. **(E)** mRNA level of *Cd68* as representative marker for monocytes/macrophages.

First, we decided to evaluate the growth of murine PHEO in young and aged mice. The mice were subcutaneously transplanted with mouse MTT PHEO cells and the development of tumors was monitored. After 46 days, the incidence of tumors in aged mice was 88.2% compared to 70% in young mice. The tumors in aged mice (n=17) were significantly larger, averaging 499.81 *±* 152.55 mm^3^ and ranging from 53.25-2020.19 mm^3^, compared to the tumors in young mice (n=20), averaging 181.83 *±* 71.76 mm^3^ and ranging 5.38-1000.43 mm^3^ in tumor volume (p=0.031, Mann-Whitney test) (**Figure 1A**). Similar trend of tumor size differences in young and old animals was also observed in bilateral pancreatic adenocarcinoma, representing another hard-to-treat tumor model with high tumor burden (**Supplementary Data**, **Supplementary Figure 4**).

Given the faster growth in our aged mice and age-related changes in number of subpopulations of T cells previously reported by other groups, we next assessed T cell markers *Cd3e*, *Cd4*, and *Cd8a* in tumors from aged and young mice (n=6/group). Quantitative PCR showed that the expression levels of these markers were extensively upregulated in young mice compared to aged mice (*Cd3e*, p=0.009; *Cd4*, p=0.041; *Cd8a*, p=0.041, Mann-Whitney test) (**Figures 1B, D**). However, when we compared the size of the analyzed tumors and theirs Cd3e mRNA expression level, we observed that lower Cd3e mRNA level is associated with increasing tumor size (p=0.013, Spearman correlation test) (**Figure 1B**). Immunohistochemical analysis of CD3 in tumors (n=6/group) confirmed the significantly higher infiltration of these cells in tumors from young mice compared to tumors from aged mice (p=0.011, Mann-Whitney test) (**Figure 1C**). Immunohistochemical analysis of CD45, as a marker of all leukocytes, showed no significant differences between young and aged mice (data not shown). Representative pictures for CD3 and CD45 are shown in **Supplementary Figures 1, 2**, respectively. We also assessed Cd68 mRNA level in tumor as a marker for monocytes, including circulating and tissue macrophages (26). Our results showed that there was no difference in Cd68 mRNA level between tumors from young and aged mice (p=0.093, Mann-Whitney test) (**Figure 1E**).

Collectively, these data point out that incidence of murine PHEOs after the subcutaneous injection of MTT cells is higher and their growth is significantly faster in aged mice than in young mice where we observed significantly higher level of *CD3e* also confirmed by IHC analysis of CD3. However, such difference was correlated with the size of tumors.

### 3.2 Efficacy of intratumoral MBTA therapy of murine PHEO in young and aged mice

**Figure 2:**
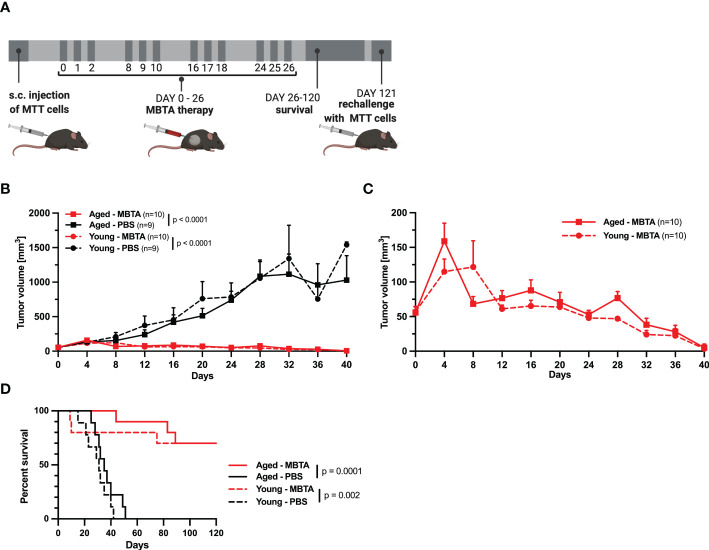
The efficacy of MBTA therapy in young (6 weeks) and aged (71 weeks) mice bearing PHEO tumor. **(A)** The schema of experimental treatment in young and aged mice. After development of tumors, the mice were randomized into treatment groups and treated on specific days. Following the 120 days for survival analysis, the mice with complete elimination of tumors were re-challenged with s.c. injection of MTT cells. **(B)** The tumor growth for all groups. **(C)** The tumor reduction for the groups of young and aged MBTA treated mice. **(D)** The survival analysis of MBTA and PBS treated mice. Days on x axis represent the days from beginning the therapy.

To evaluate the age-dependent effect of MBTA therapy, we applied MBTA therapy into subcutaneous PHEO tumors of young and aged mice. First, the mice were subcutaneously injected with MTT cells. Therapy of aged mice started on day 23 after the injection of MTT cells when tumors were averaging 55.92 ± 6.27 mm^3^ (23.44-114.96 mm^3^). For young animals, therapy started on day 30 when tumors were averaging 54.65 ± 5.25 mm^3^ (24.8-115.02 mm^3^) (**Figure 2A**). The growth of PHEOs confirmed the faster growth in aged mice compared to young mice observed in first experiment. The reduction of tumor growth was similar in aged and young MBTA treated mice compared to control group (aged, p<0.0001; young, p<0.0001) (**Figures 2B, C**). Both aged and young MBTA treated mice demonstrated significant increase in survival compared to their control groups (aged, p<0.0001; young, p=0.002, Log-rank test) (**Figure 2D**). No significant difference was observed in reduction of tumor growth or survival between aged and young MBTA treated mice. The experiment was done twice with comparable outcomes (**Supplementary Figure 3**) This observation was also confirmed in bilateral Panc02 model where the reduction of tumor growth during MBTA therapy was similar between aged and young mice (**Supplementary Data**, **Supplementary Figure 4**).

To assess the efficacy and immune memory in both aged and young animals, the mice were followed up for possible recurrence and re-challenged with transplantation of PHEO cells. In post-treatment observation of 120 days, 7/10 of aged or young MBTA treated mice bearing PHEO achieved complete remission for the duration of study. On day 121, these mice were retransplanted again with the same number of MTT cells as described. All mice remained tumor free after 40 days from re-transplantation suggesting a long-term immunological memory after MBTA therapy (data not shown).

## 4 Discussion

Over the past years, immunotherapy has become one of alternative to conventional cancer treatments. Patients have benefited from inhibition of checkpoint inhibitors, especially programmed cell death protein 1/ligand 1 (PD-1/PD-L1) and cytotoxic T-lymphocyte-associated protein 4 (CTLA-4) (27). However, these immunotherapeutics are administered systemically which can be associated with off-target and dose-related toxicities (28). Contrary, intratumoral injection enables to use much lower dosage of immunotherapeutics and thus lower systematic exposure (29). This advantage makes intratumoral administration a feasible candidate for cancer treatment in elderly patients who may have age-related immune dysfunction, collectively called immunosenescence.

A mouse model is commonly used animal model for investigation of immune system as well as for immunosenescence. Many age-related alterations in immune system have been described in both mice and humans. Changes in innate immunity include decreased phagocytosis by neutrophils, antigen presentation by macrophages and dendritic cells, and cytokine and chemokine productions (30, 31). Contrary, changes in adaptive immune cells are associated with decreased development of naïve T cells, but increased number of memory specific T cells and regulatory T cells (32, 33). In case of B cells, increased production of autoantibodies and decreased immunoglobulin production have also been described in elderly compared to young patients (34).

In the present study, we compared the growth of subcutaneous PHEO in 6-weeks (young) and 71-weeks (aged) old mice with subsequent application of intratumoral MBTA therapy to better mimic the age of patient with PHEO which is an important factor for potential use of this approach in future clinical trial. We showed that the incidence of subcutaneous pheochromocytoma tumors is higher in aged mice, and the growth of PHEO are faster in aged mice compared to young mice. Published studies have previously shown the difference in tumor growth in aged and young mice with general acceptance of slower growth in aged mice (35, 36). However, for example, murine mesothelioma AE17 (37), prostate adenocarcinoma TRAMP-C2 (38), and colon carcinoma CT26 (39) models have showed faster growth in aged mice compared to young mice, suggesting the specificity for each tumor cell line. Indeed, in our second model, bilateral Panc02 tumor model which here better represents the real situation in patient with higher tumor burden, we observed significantly larger tumor volumes in 72-weeks (aged) animals compared to in 8-weeks (young). Previously, age-related changes in number of subpopulations of T cells have been widely reported before and T cells may have a key role in tumor development in general. In case of PHEO, we measured higher levels of mRNA of *Cd3e*, *Cd4*, and *Cd8a* in tumors from young mice, as well as CD3+ positive cells, suggesting higher infiltration of T cells in the tumors. However, such higher level of *Cd3e* negatively correlated with size of analyzed tumors between groups, suggesting that the infiltration is based on size of tumors and not age difference.

Intratumoral MBTA immunotherapy of PHEO showed no age-related differences in efficacy and was able to completely reduce tumor growth in 70% of treated both young and old animals. Such results are consistent with our previous results with young mice bearing pheochromocytoma, pancreatic adenocarcinoma, melanoma, or colon carcinoma where MTBA immunotherapy was efficient in 62-83% (14–16). Similarly, the previous studies of the efficacy of MBTA in pancreatic adenocarcinoma, Panc02 tumor model (15, 17), were done in young animals only while pancreatic cancer most often affects older adults (40). Our results of MBTA in bilateral Panc02 tumor model, which was tested in 8-weeks and 72-weeks old mice, representing 15 years and 57 years in human, showed the similar reduction of tumors and prolonged survival of treated mice as published before (17). Such results suggest that MBTA therapy is effective even in this hard-to-treat Panc02 model with two advanced tumors without any age differences. While in PHEO model the survival was comparable for both age groups, we observed the difference in survival of MBTA-treated mice between aged (1/6 mice) and young mice (3/6 mice) in Panc02 model. Such difference can be explained by different tumor size at the beginning of therapy when the tumors in aged mice were significantly bigger than in young mice.

Interestingly, despite increasing interest in intratumoral application, there is limited number of studies comparing the efficacy of intratumoral immunotherapy in young and old animals. Sharma et al. compared the intratumoral application of poly(I:C) and CpG-ODN in 2-4-months and 18-22-months old mice. Their results indicated that only intratumoral injection of CpG-ODN induced the complete rejection of mammary tumors (TUBO) and delayed prostate adenocarcinoma tumor (TRAMP-C2) growth in both young and old mice. Contrary, intratumoral injection of poly(I:C) induced the rejection of TUBO tumors and delayed the growth of TRAMP-C2 tumors in young but not in the old mice (41). Another example of study focused on an efficacy of intratumoral immunotherapy in young and aged mice was published by Duong et al. (37). They compared the efficacy of IL-2/anti-CD40 antibody and founded this therapy less effective in aged (38%) than in young mice (90%). Even though it appears that the tolerance of the tumor growth and progression of murine PHEO in aged and young mice were different, both age groups have a similar immune response once triggered by intratumoral application of MBTA.

Although clinical trials have not showed major increases in age-related adverse events during checkpoint inhibitor therapies (42), it should be mentioned that there is still limited evidence due to underrepresentation of elderly patient in clinical trials (42, 43). While we did not observe any adverse events during MBTA immunotherapy in aged mice, 2 young mice were found dead on day 10 (after the second cycle of MBTA therapy). However, the cause of death is momentarily unknown. We assume that the cause of death may be related to the extensive activation of immune system and weight of animals which is not comparable between young and aged mice. This limitation of study should be answered in the future for further and safe use of MBTA immunotherapy.

Incidence of tumor development is continually increasing with age of patients. However, many preclinical studies are not focused on age-related differences and clinical studies do not often include elderly patients. Here, we demonstrate that intratumoral MBTA immunotherapy has same efficacy in young and old PHEO-bearing mice. However, future clinical study is necessary to confirm the efficacy of described intratumoral immunotherapy in patients.

## Data availability statement

The raw data supporting the conclusions of this article will be made available by the authors, without undue reservation.

## Ethics statement

The animal study was reviewed and approved by Animal Care and Use Committee of the National Institutes of Health (ASP 18-028, pheochromocytoma model) and Ministry of Education, Youth, and Sport of the Czech Republic (Protocol No. 12098/2016-2, pancreatic adenocarcinoma model).

## Author contributions

OU, KHV, JZ, and KP designed the study. OU, KHV, RL, and AF performed the experiments. OU, KHV, and HW verified, analyzed, and interpreted the data. JZ, ZZ, and KP supervised and administrated the project. All authors wrote, read, and approved the final manuscript.
