# Supplementary material for: Intratumoral immunotherapy of murine pheochromocytoma shows no age-dependent differences in its efficacy
Source: Front Endocrinol (Lausanne). 2023 May 8;14:1030412. doi: 10.3389/fendo.2023.1030412 (PMC10277857; doi:10.3389/fendo.2023.1030412)
Supplement: Supplementary file 1 [file DataSheet_1.docx]

Supplementary Material – ****Intratumoral Immunotherapy of Murine Pheochromocytoma Shows No Age-Dependent Differences in Its Efficacy****

Ondrej Uher, Katerina Hadrava Vanova, Radka Lencova, Andrea Frejlachova, Herui Wang, Zhengping Zhuang, Jan Zenka, and Karel Pacak

# Supplementary methods

## **Panc02 cell line, mice, tumor model establishment, and tumor size evaluation**

The murine pancreatic adenocarcinoma cell line (Panc02) was obtained from Prof. Lars Ivo Partecke (Greifswal, Germany). Cells were cultured at 37 °C in humidified air with 5% CO_2_. Cell lines was tested for mycoplasma using the MycoAlert™ detection kit purchased from Lonza (Walkersville, MD, USA). Female C57BL/6N mice were purchased from Charles River, Laboratories (Sulzfeld, Germany). The experiment was performed on young (8 weeks) and aged (72 weeks) mice. Mice were housed in specific pathogen-free barrier facilities with free access to sterile food and water, the photoperiod was 12/12. To establish bilateral Panc02 model, mice were subcutaneously injected with 4 × 10^5^ Panc02 cells in 0.1 mL of DMEM without additives into previously shaved right and left lower dorsal sites. Tumor volumes, survival curves, and MBTA therapy were followed as listed in Material and Methods for PHEO model.

# Supplementary results

## **Growth of subcutaneous murine bilateral pancreatic adenocarcinoma tumor model in young and aged mice and efficacy of intratumoral MBTA therapy in this model**

The incidence of tumors was 100% in aged and young animals after 12 days from the bilateral transplantation. The tumors in aged mice (n=10 mice/20 tumors) were averaging 80.48***±***9.53 mm^3^ and ranging from 27.57-175.31 mm^3^ which were significantly larger compared to the tumors in young mice (n=12 mice/24 tumors) averaging 50.65±4.61 mm^3^ and ranging from 30.29-108.77 mm^3^ in tumor volume (p=0.028, Student’s t-test) (Suppl.Fig. 4A). Therapy started on the day 12 and was intratumorally applied into both, right and left, tumors (Suppl.Fig. 4B). The reduction of tumor growth during MBTA therapy was similar in aged and young mice compared to control groups (young – right tumors, p<0.001, young – left tumors, p<0.0001, aged – left tumors, p=0.0093) (Suppl.Fig. 4C, D) except for the right tumors of aged mice where we did not observe statistical significance (p=0.2072). Young MBTA-treated mice demonstrated significant increase in survival compared to their control group (p=0.0072) and resulted in complete regression in 3/6 of mice (Suppl.Fig. 4E). MBTA therapy in aged mice resulted in complete regression in 1/6 of treated mice, however, statistical significance for survival was not reached when compared to the control group (p=0.0633). There was no significant difference between aged and young MBTA-treated mice (p=0.0807) but different trend in the survival of aged animal is obvious.

# Supplementary Figures

**Suppl. Fig.1** Representative pictures from CD3 staining in pheochromocytoma tumors from aged and young mice (n=7/group). Bar showing 200 μm, zoom 10x.

**Suppl. Fig.2** Representative pictures from CD45 staining in pheochromocytoma tumors from aged and young mice (n=7/group). Bar showing 200 μm, zoom 10x.

**
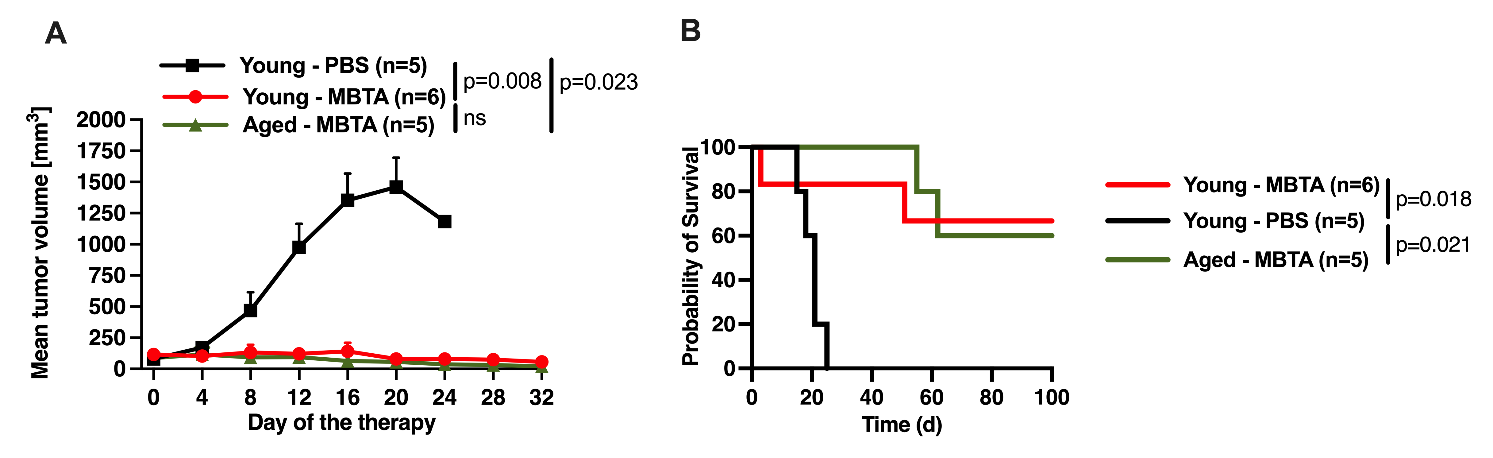
**

**Suppl. Fig.3** The efficacy of MBTA therapy in young (8 weeks) and aged (67 weeks) mice bearing pheochromocytoma tumor – additional conformation experiment. Methodology of the experiment was same as described in main text. *Note:* Mice for the aged group were housed in our animal facility until they reached appropriate age and not bought from JAX as animals who were included in the main experiment.


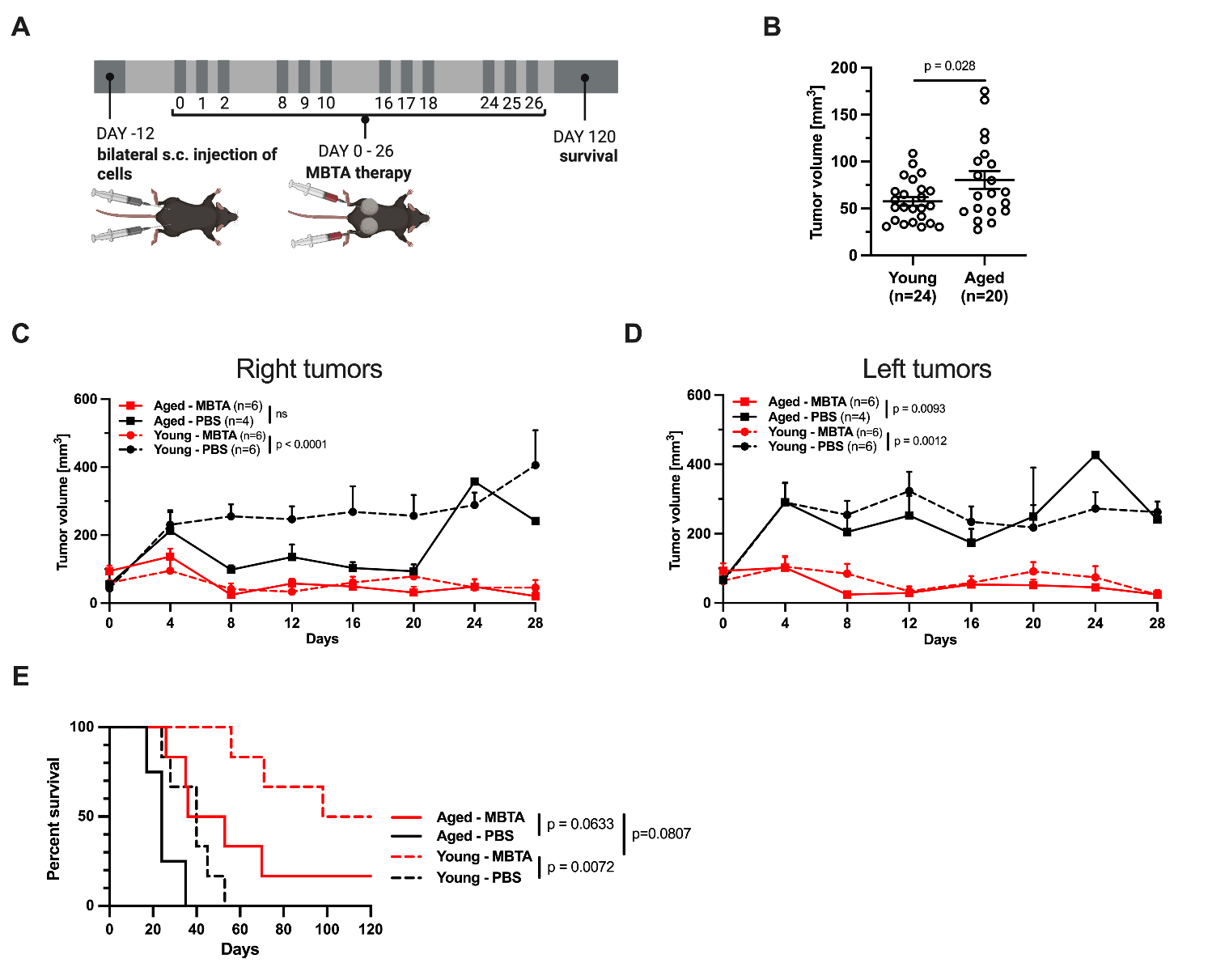


**Suppl. Fig.4** The efficacy of MBTA therapy in young (8 weeks) and aged (72 weeks) mice bearing bilateral Panc02 tumor model. **A** The schema of experimental treatment in young and aged mice. On day 12 from s.c. transplantation of Panc02 cells, the mice were randomized into treatment groups and treated on specific days following the 120 days for survival analysis. **B** The tumor volume in young and aged mice on day 12. **C** The tumor growth for right tumors. **D** The tumor growth for left tumors **E** The survival analysis of MBTA and PBS treated mice. Days on x axis represent the days from beginning the therapy.

# Supplementary Table

**Supplementary Table 1:** Forward and reverse primers.

| Gapdh | Forward | 5’-GCCTTCCGTGTTCCTACCC-3’ |
| --- | --- | --- |
|  | Reverse | 5’-CAGTGGGCCCTCAGATGC-3’ |
| Cd3e | Forward | 5’-GCTCCAGGATTTCTCGGAAGTC-3’ |
|  | Reverse | 5’-ATGGCTACTGCTGTCAGGTCCA-3’ |
| Cd4 | Forward | 5’-GTTCAGGACAGCGACTTCTGGA-3’ |
|  | Reverse | 5’-GAAGGAGAACTCCGCTGACTCT-3’ |
| Cd8a | Forward | 5’-ACTACCAAGCCAGTGCTGCGAA-3’ |
|  | Reverse | 5’-ATCACAGGCGAAGTCCAATCCG-3’ |
| Cd68 | Forward | 5’-GGCGGTGGAATACAATGTGTCC-3’ |
|  | Reverse | 5’-AGCAGGTCAAGGTGAACAGCTG-3’ |
